# Evolutionary Dynamics of Avian Influenza Viruses Isolated from Wild Birds in Moscow

**DOI:** 10.3390/ijms24033020

**Published:** 2023-02-03

**Authors:** Yulia Postnikova, Anastasia Treshchalina, Alexandra Gambaryan, Alla Belyakova, Aydar Ishmukhametov, Mikhail Matrosovich, Galina Sadykova, Alexey Prilipov, Natalia Lomakina, Elizaveta Boravleva

**Affiliations:** 1 Department of Virology, Faculty of Biology, Lomonosov Moscow State University, 119991 Moscow, Russia; 2 Chumakov Federal Scientific Center for the Research and Development of Immune-and-Biological Products, Village of Institute of Poliomyelitis, Settlement “Moskovskiy”, 108819 Moscow, Russia; 3 Institute of Virology, Philipps University, Hans-Meerwein-Str. 2, 35043 Marburg, Germany; 4 The Gamaleya Research Center for Epidemiology and Microbiology of the Russian Ministry of Health, 123098 Moscow, Russia

**Keywords:** avian influenza, genome dynamics, reassortment

## Abstract

Forty-five strains of AIVs were isolated from wild aquatic birds during their autumn migration through Moscow (Russia). The aim of this work is to study the dynamics of AIV genomes in their natural habitat. Viruses were isolated from fecal sample in embryonated chicken eggs; their complete genomes were sequenced, and a phylogenetic analysis was performed. The gene segments of the same lineage persisted over the years in the absence of persistence of complete viral genomes. The genes for internal proteins of the same lineage were often maintained by the viruses over few years; however, they were typically associated with the genes of novel HA and NA subtypes. Although frequent reassortment events were observed for any pair of internal genes, there was no reassortment between HA and NA segments. The differences in the persistence of phylogenetic lineages of surface and internal proteins and the different evolutionary strategy for these two types of genes of AIVs in primary hosts are discussed.

## 1. Introduction

Influenza virus (IV) is an RNA virus with a negative-sense single-strand RNA genome. The genome contains 8 RNA segments encoding 10 main and several accessory proteins depending on the strain. The pool of influenza A viruses includes 18 HA subtypes and 11 NA subtypes; among them subtypes H17N10 and H18N11 were found only in bats [1].

Metagenomic studies suggest that IVs evolved in vertebrates for millions of years [2]. Wild birds of the order *Anseriformes* are the primary hosts of influenza A viruses. In these hosts, the virus replicates in the lower intestine causing asymptomatic infection [3,4]. Most birds become infected with avian IV (AIV) in the first months of life. Shedding of the virus into the environment (water) occurs within a few days after infection [5]. The host develops an immune response. In 60–76% of mallards of the first year of life and in 86–93% of mallards hatched a year earlier, antibodies to AIV were detected. Most NP-seropositive ducks contained antibodies to multiple HA and NA subtypes [6]. The molar ratio in the virion of the four major structural proteins HA:NA:NP:M1 is approximately 26:3:22:100, respectively [7]. However, antibody responses to HA, NA, and NP were 55%, 35%, and 10%, respectively, while the response to M1 was negligible [8].

AIVs of different subtypes can co-circulate among waterfowl in their natural habitats. AIV genomes are constantly changing due to gene reassortments [9,10]. The reassortment rate is particularly high in low pathogenic avian influenza viruses (LPAIV) [11]. As a result, AIVs in wild birds do not circulate as permanent genome constellations, but as a pool of interchangeable gene segments that form temporary constellations [12].

New evolutionary lineages usually emerge in new hosts following a successful reassortment. All pandemic human influenza A viruses and many lineages of highly pathogenic chicken viruses originated this way [4]. In the course of evolution, many phylogenetic lineages of influenza viruses disappeared and were replaced by new lineages. Thus, the 2009 H1N1 pandemic virus forced the previous H1N1 virus out of circulation. It is possible that prolonged circulation of viruses in immune hosts is associated with fitness costs [13]. In the second half of the 18th century, an avian influenza virus (probably H7N7) initiated a global sweep of gene segments of internal viral proteins of IV, replacing almost all previous gene variants [14].

There is a contrast between the evolutionary history of the gene segments encoding surface glycoproteins of influenza viruses (HA and NA) and the other gene segments. Modern IVs contain variants of surface glycoproteins that evolved for over thousand years, whereas all internal IV genes, with the exception of the NS1/2 gene segment and gene segments of bat IVs, had a common ancestor in the second half of the 19th century. Moreover, global sweeps continued into the 20th century. The PB1, PA, NP, and NS genes of modern Eurasian IVs and almost all American IVs are descendants of poultry AIV of the 1920s–1930s, while older gene lineages have almost completely disappeared. It is important that genes are transferred not only from wild bird viruses to poultry viruses, but also in the opposite direction [14].

Studying the circulation of avian influenza viruses in the wild is essential to monitor and prevent transmission of AIV between wild and domestic birds. Since 2006, we regularly isolated AIVs during the autumn migration of wild birds through Moscow (Russia). Forty-five of the isolated strains were completely sequenced [15]. The aim of this work is to study the dynamics of genomes in their natural habitat by analogy with the work of Dugan and colleagues [12].

## 2. Results

**Figure 1:**
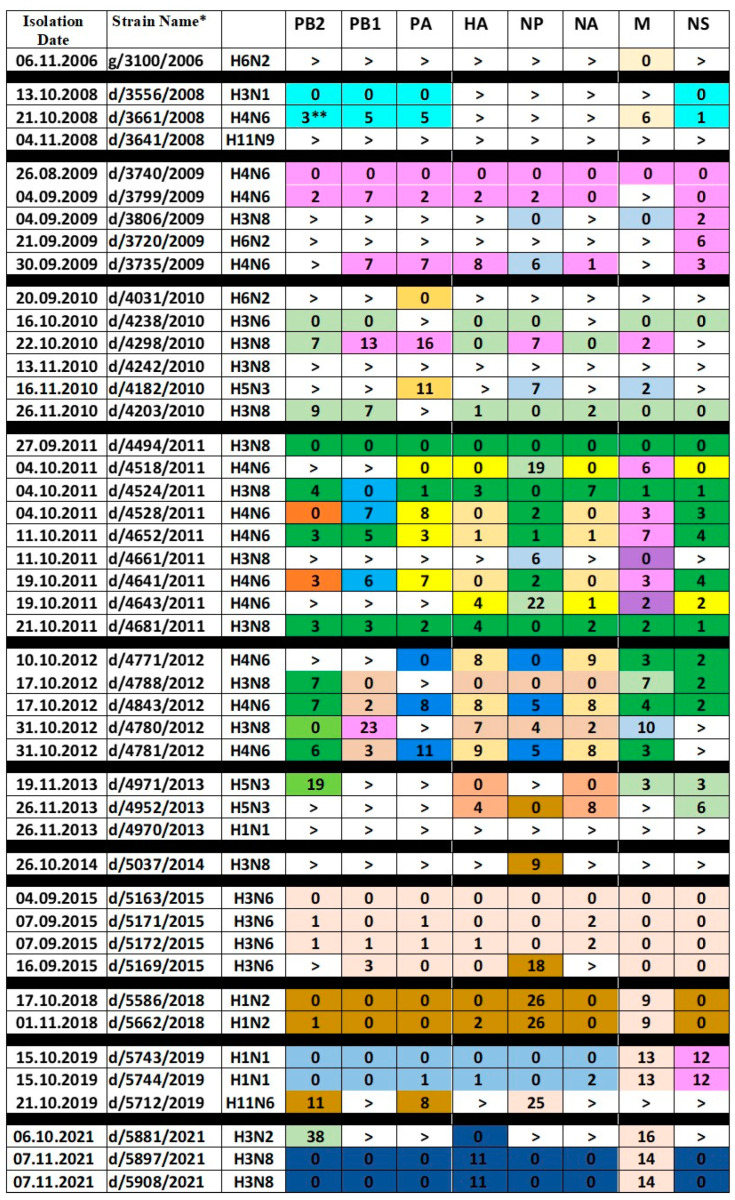
Genome variants of IAVs isolated in Moscow. * The viruses are designated by host (g—gull and d—duck), strain number and isolation year. For example, d/5743/2019 stands for A/duck/Moscow/5743/2019. ** The number in the cell shows the number of nucleotide substitutions with respect to the earlier gene of the lineage marked as “0”. Sign “>” indicates that the gene segment in this cell differed from all other genes in the column by more than 1% of nucleotide substitutions. The same color of the cells in the column depicts gene segments sharing more than 99% homology.

From 2006 to 2021, fecal samples of wild gulls (*Larus ridibundus*) and mallards (*Anas platyrhynchos*) were collected on Moscow ponds to identify avian influenza viruses. The sampling was performed during autumn migration of birds from September to November. The analysis of the genomes is presented in the Figure 1. The table was constructed as described in Materials and Methods. In brief, viruses were ordered in accord with the time of their isolation. The gene segment marked with the sign “>” differed from all corresponding segments of IVs isolated at a later data by more than 1% of nucleotide substitutions. If the segment shared more than 99% homology with the subsequent segments, corresponding cell of the table was colored and marked as “0”, indicating the earliest occurrence of the genetic lineage in the analyzed virus set. Subsequent segments of this lineage were labeled by the same color. The numbers of nucleotide substitutions with respect to the first sequence of the group were indicated in the cells. The number of substitutions increased over the years from 1–2 up to 10–30. Thus, we could trace the phylogenetic lineage of the gene segment over several years.

The only virus isolated in 2006, g/3100/2006 (H6N2), is unique in all gene segments, except for M. The M gene, which differs by only six nucleotide substitutions, was found in the d/3661/2008 (H4N6) virus in 2008.

Three viruses from 2008 have different HA, NA, NP and MP genes. However, in two isolates, d/3556/2008 (H3N1) and d/3661/2008 (H4N6), the PB2, PB1, PA, and NS genes are very similar. The d/3661/2008 (H4N6) virus is a triple reassortant. Its PB2, PB1, PA, and NS genes are related to the corresponding genes of the d/3556/2008 (H3N1) virus, the HA, NP, and NA genes are derived from an unknown virus, and the M gene is related to the M gene of g/3100/2006 (H6N2).

The three 2009 H4N6 viruses have nearly identical PB1, PA, HA, NA, and NS genes. However, their M genes are different, and d/3735/2009 (H4N6) also has different PB2 and NP genes. Two other viruses of 2009, d/3806/2009 (H3N8) and d/3720/2009 (H6N2) differ from H4N6 viruses in all genes, except for NS, which is close to each other in all five isolates and belong to allele B. That is, all the 2009 isolates resulted from a reassortment with a virus carrying a common NS gene and subsequent reassortments with the H4N6, H3N8, and H6N2 viruses. The NP genes of d/3735/2009 (H4N6) and d/3806/2009 (H3N8) viruses are related to each other.

In 2010, the H6N2, H3N6, H3N8, and H5N3 viruses were isolated. The d/4031/2010 (H6N2) virus, like the two previous H6N2 viruses, is unique in almost all genes, with the exception of the PA gene, which differs from the PA of the d/4182/2010 (H5N3) virus by 11 nucleotides. The d/4203/2010 (H3N8) virus is very similar to the d/4238/2010 (H3N6) virus, but has the different variant of PA gene and the different neuraminidase, which it inherited from the d/4298/2010 (H3N8) virus. The d/4182/2010 (H5N3) virus is unique in five genes, except for PA, NP, and M, with the last two genes being descendants of the corresponding genes of the d/3806/2009 (H3N8) virus. The H5N3 viruses of 2010 and 2013 differ in all gene segments. Interestingly, two H5N3 viruses from 2013 contained three highly homologous segments and five other segments with very low homology.

Sometimes the viruses had genes inherited from isolates of previous years. So, three strains of 2011 inherited the M gene from viruses of 2009 and 2010. In 2012, H4N6 viruses inherited the PB2, HA, NA, M, and NS genes from the 2011 viruses. The d/4971/2013 (H5N3) virus contained the M and NS genes, which differed from the corresponding genes of 2010 by only 3 nucleotide substitutions. It can be noted that almost all internal genes inherited from isolates of the previous year were found in the viruses with HA/NA of a novel subtype.

Analysis of the Figure 1 reveals the following patterns:

1. The exact genomic profile of the virus as a whole has never been preserved in subsequent years.
2. Genes closely related to those found in viruses earlier occur in subsequent years, with a gradual accumulation of substitutions. For example, compared to respective segments of d/3740/2009 (H4N6), PB1 of d/4780/2012 (H3N8) accumulated 23 nucleotide substitutions over three years of circulation, and NS of d/5743/2019 (H1N1) accumulated 12 nucleotide substitutions over 10 years of circulation.
3. The genes for internal proteins, which were preserved in the viruses on the next year of isolation, as a rule were not associated with the NA and HA of the same subtype. Thus, the M gene of d/3661/2008 (H4N6) was inherited from g/3100/2006 (H6N2), the PB1, PA, NP, and M genes of d/4298/2010 (H3N8) were inherited from d/3740/ 2009 (H4N6), while the NP and M genes of d/4182/2010 (H5N3) were inherited from d/3806/2009 (H3N8).
4. The dynamics of internal genes is fully consistent with the pattern described by Dugan et al.: «AIV in wild birds exists as a large pool of functionally equivalent, and so often interchangeable, gene segments that form transient genome constellations, without the strong selective pressure to be maintained as linked genomes» [12]. Interclade reassortment events have been observed for any pair of internal genes.
5. Nevertheless, the genes of the surface proteins (HA and NA) evolved as a pair. If one of them was replaced by a variant from another clade, then the second gene was also replaced. Thus, in 12 H4N6 viruses and in 13 H3N8 viruses, we did not find a single case of separation of the HA-NA pair. At the same time, pairs of hemagglutinin with any of the other genes were not stable and changed as the internal genes were reassorted (Table 1).
6. A total of 25 out of 45 Moscow isolates belong to the dominant AIV subtypes, H3N8 and H4N6. Other combinations, such as H3N1, H3N2, H3N6, also occurred, but much less frequently (Table 2).

In order to test whether observed restrictions on reassortment between HA and NA segments are real, or they are accidental and can be explained by the limited size of our data, we analyzed larger panels of available AIV sequences. In this work we focused on sets of H4N6 and H3NX viruses from EpiFlu GISAID [16] and Influenza Research Database [17].

**Figure 2:**
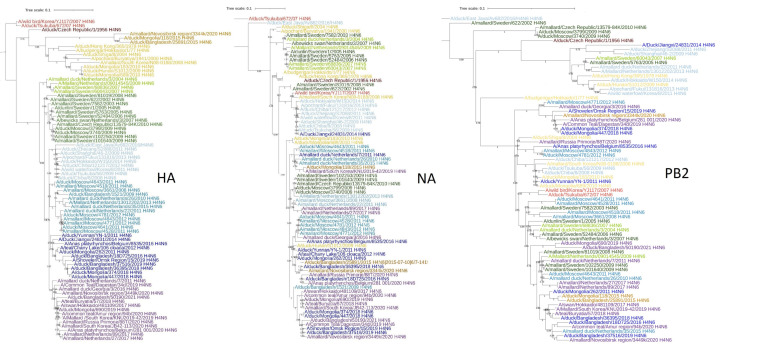
Phylogenetic tree of the HA, NA, and PB2 genes of Eurasian H4N6 viruses. The viruses are painted with the same color on different trees. Scalable versions of the trees are given in Supplementary Materials, Figures S1–S3.

The evolutionary trees of the HA, NA, and PB2 genes of Eurasian H4N6 viruses are shown in Figure 2 and Figures S1–S3 of Supplementary Materials. The structure of the evolutionary trees of the HA and NA genes is similar. For example, almost all viruses are congruent on branches that include duck/Chiba/8/2008 and duck/Hokkaido/W150/2014. Obviously, from 2008 to 2014, the HA and NA of these viruses co-evolved. However, 17 cases of interclade reassortment between the HA and NA genes can be identified on these trees. The evolutionary trees of the HA and PB2 genes are less congruent. In this case we identified 54 cases of interclade reassortment between the HA and PB2 genes.

A similar result was obtained in the analysis of wild duck viruses isolated in the USA from 2010 to 2020. Interclade reassortment between the HA and NA genes occurred less frequently than between HA and PB2, but, nevertheless, quite intensively (Table 3 and Figures S4–S6 of Supplementary Materials).

Analysis of the IV of H3 subtype revealed numerous cases of reassortment both with neuraminidases of other subclades and with neuraminidases of the N1, N2, and N6 subtypes (Table 3 and Figures S7–S9 of Supplementary Materials). All these examples show that reassortment between the HA and NA genes in wild bird AIVs occurs quite regularly, both between different clades of these genes, and sometimes with another subtype of neuraminidase. However, “canonical” HA-NA combinations continue to dominate in the population. The number of viruses with different combinations of HA and NA subtypes among viruses isolated from wild *Anseriformes* in the United States in 1990–2020 is shown in Table 4.

In general, these results agree with the data of Liu et al., who performed similar analyses of all known subtypes of influenza A viruses [18]. As we only considered viruses isolated from wild *Anseriformes*, fewer combinations were observed in this set. Combinations of H3N8 and H4N6 dominated; although combinations of the same HA subtypes with different NA subtypes such as H3N2, H3N6, H4N2, and H4N8 were also present. The proportion of latter non-canonical pairs approximately corresponded to the prevalence of corresponding NA subtypes in general.

## 3. Discussion

Several potential mechanisms may determine the dominance of specific combinations of HA and NA genes:

(1) Co-evolution of HA and NA pairs of genes without reassortment.
(2) Structure of viral RNA segments of specific subtypes being adapted to each other [19,20].
(3) Characteristics of viral proteins that provide a functional balance of NA and NA [21,22].

Analysis of sequence databases argues against the first hypothesis as frequent interclade and intersubtype reassortment between the HA and NA genes can be observed.

It is possible that the second mechanism ensures the sustainable combination of the rare subtypes such as H8 and N4 or H12 and N5; however, presence of a substantial amount of viruses with “non-canonical” combinations is not consistent with this mechanism for dominant subtypes.

According to the third mechanism, newly emerging reassortant viruses would have reduced viability until compensatory mutations appeared in the HA and/or NA genes. However, in our study, H3 subtype viruses with different NAs did not seem to differ in infectivity, invasiveness, and virulence. Thus, strains d/4238/2010 (H3N6) and d/4298/2010 (H3N8), isolated almost simultaneously in one place, did not have a single nucleotide substitution in HA, but were equally productive in chicken embryos, successfully infected mice and chickens, causing a pronounced immune response. In addition, H3N1 and H3N2 viruses were not distinguished by these parameters [23]. Although the limited amount of our data does not allow drawing of formal conclusions, our results seem to indicate that gene reassortment with the formation of a non-canonical pair of HA H3 and NA results in emergence of a viable virus, but then it is swept away by natural selection.

This may occur during the circulation of the virus in wild birds. The short life cycle of the virus and the intensity of infection within a flock make it difficult for the virus to persist until the next season [6]. A strong immune response against surface proteins prevents reinfection of the bird with the same virus, but a weak response to internal proteins allows infection with viruses of other subtypes [8]. Autumn migration and large local concentrations of waterfowl during wintering provide the possibility of secondary infection with AIVs with HA and NA of other subtypes [6,24].

The infections with reassortant virus that have retained some or all of the internal proteins from the first virus is also possible. Internal genes would benefit from the reassortment with acquisition of the new segments encoding antigenically novel surface proteins. This phenomenon enables a strong competition between the genes of internal proteins for antigenically novel HA and NA. In AIVs of wild birds a winning evolutionary strategy for genes of internal proteins is the ability to combine with any variants of all other genes. This strategy is fully consistent with the phenomenon described by Dugan et al.: “gene segments form transient genomic constellations without selective pressure to be maintained as linked genomes” [12].

Contrariwise, the unlimited combination of surface proteins complicates reinfection. Primary infection with the HXNY variant creates an immune barrier for all viruses containing either HX or NY. For example, H4N8 reassortants cannot reinfect hosts that have previously been infected with both H4N6 and H3N8 viruses. Once the dominance of certain combination has been established, it will be self-maintained. Thus, H4N6 viruses can re-infect hosts that were previously infected with H3N8 and vice versa. At the same time, H4N8 and H3N6 reassortants will encounter restricting immune pressure against HA and/or NA in all hosts, previously infected with either H4N6 or H3N8 viruses, the most dominant variants.

The evolutionary strategy of wild AIVs includes competition of internal genes with constantly updated combinations and regular replacement of a pair of surface proteins. The new successful variant of internal gene displaces the previous variant from circulation. By contrast, different subtypes of surface proteins do not compete with each other. If the proportion of some antigenic variant decreases, it gains an advantage and can be expanded. The alternation of surface antigens supports the circulation of AIV in general. These mechanisms may explain the differences in the persistence of phylogenetic lineages of surface and internal proteins [14].

It is important to emphasize that the pattern described above is only typical for the IVs of the primary hosts, wild ducks. With the formation of new evolutionary lineages in other hosts the dynamics of the genome changes. When adapting to a new host, the rate of mutations in all genes increases and a genome constellation is fixed, which subsequently evolves as a whole [25]. Even in wild gulls, long-lasting lineages of H13N6 and H16N3 viruses were formed, which did not reassort with wild duck viruses and rarely reassorted with each other [26]. In chickens, influenza virus lineages have evolved as a whole for years; in pigs, horses, and humans, they have evolved for decades.

## 4. Materials and Methods

### 4.1. Reagents

Viral RNA Mini Kit was from QIAGEN, Hilden, Germany. MMLV Reverse Transcription kit, random primers, nuclease free water, DNA ladder, and TAE buffer were from Evrogen, Moscow, Russia. MycoKill AB solution was from PAA Laboratories GmbH, Pasching, Oberosterreich, Austria.

### 4.2. Viruses

Fresh feces of birds were collected in 2006–2021 on the shore of the ponds in Moscow. Feces were suspended in a double volume of phosphate-buffered saline supplemented with 0.4 mg/mL gentamicin, 0.1 mg/mL kanamycin, 0.01 mg/mL nystatin, and 2% MycoKill AB solution. The suspension was centrifuged for 10 min at 4000 rpm, and 0.2 mL of the supernatant was inoculated into 10-day-old chicken embryos. Infected allantoic fluid was collected after 48 h and tested by hemagglutination assay with chicken erythrocytes. All strains were deposited in the virus repository of Chumakov Federal scientific center for the research and development of immune-and-biological products, Moscow, Russia. Full names, designations of the viruses and GenBank accession numbers are given in Table S1 of Supplementary Materials.

### 4.3. Sequencing 

Viral RNA was isolated from the allantoic fluid of infected chicken embryos with a commercial QIAamp Viral RNA mini kit (QIAGEN, Hilden, Germany). Full-length viral genome segments were obtained by reverse transcription and PCR with specific terminal primers, MMLV, and Taq-polymerase (Alpha-Ferment Ltd., Moscow, Russia). The amplified fragments were separated by electrophoresis in 1–1.3% agarose gel and extracted from the gel with the Diatom DNA elution kit (Isogene Laboratory Ltd., Moscow, Russia, # D1031). Sequencing reactions were performed with terminal or internal primers with the BrightDye™ Terminator Cycle Sequencing Kit v3.1 (Nimagen, the Netherlands) followed by analysis on an ABI PRISM 3100-Avant automated DNA sequencer (Applied Biosystems 3100-Avant Genetic Analyzer, Foster City, CA, USA). The Lasergene 6 software package (DNASTAR Inc., Madison, WI, USA) was used for assembly and analysis of nucleotide sequences.

### 4.4. Analysis of Genomes

Based on the complete sequences of all genes, a table was constructed (see the table in Figure 1), where each row corresponded to the isolate, and each column corresponded to a gene segment. The strains were arranged in the order in which they were isolated. The sequences of the gene segments were compared, starting with earlier isolates. The table cells corresponding to the gene segments were marked with a “>” sign if this segment differed from all its descendants by more than 1% of nucleotide substitutions. If the segment shared more than 99% homology with the descendants, corresponding cell of the table was colored and marked with a “0” sign. Subsequent segments of this lineage were labeled by the same color. The numbers of nucleotide substitutions with respect to the first sequence of the group were indicated in the cells.

### 4.5. Phylogenetic Analysis

The complete nucleotide sequences of AIV genes were downloaded from the EpiFlu GISAID [16] and Influenza Research Database [16] accessed on 20 October 2022. The selected sequences were aligned by the MUSCLE method, and maximum-likelihood trees with 1000 bootstrap replicates were built using the MEGA software v.7.0.26 [27] based on the general time-reversible (GTR) model. Evolutionary rate differences among sites were modeled with discrete Gamma distribution (G), and the rate variation model allowed for some sites to be evolutionarily invariable (I).

## 5. Conclusions

Fourty-five strains of AIV were isolated from wild birds in Moscow (Russia) and completely sequenced. Evolutionary dynamic and reassortment of gene segments were analyzed and compared with the data in the sequence databases. The persistence for several years of gene segments of internal viral proteins accompanied by a regular replacement of the genes of the surface proteins HA and NA was observed.

## Figures and Tables

**Table 1:** Number of interclade reassortments between the HA gene and other genes.

Subtype	Reassortment between the HA Gene with Genes:						
	PB2	PB1	PA	NP	NA	M	NS
H3N8	1	3	2	1	0	2	2
H4N6	4	3	2	2	0	4	1
H5N3	1	1	1	1	0	1	0

**Table 2:** Number of viruses of different subtypes among Moscow isolates.

	Subtypes										
	H1N1	H1N2	H3N1	H3N2	H3N6	H3N8	H4N6	H5N3	H6N2	H11N9	H11N6
Number	3	2	1	1	5	13	12	3	3	1	1

**Table 3:** Number of interclade reassortments between hemagglutinin and NA or PB2 genes.

Location, Subtype, (Number of Viruses)	Reassortment between HA and:	
	NA	PB2
Eurasian H4N6 (72)	17	54
USA H4N6 (450)	68	120
Eurasian H3Nx (74)	41	34

**Table 4:** Number of AIV strains with different combinations of HAs and NAs isolated from wild birds of the order *Anseriformes* in the United States in 1990–2021. Influenza Research Database (https://legacy.fludb.org, accessed on 20 October 2022).

	H1	H2	H3	H4	H5	H6	H7	H8	H9	H10	H11	H12	H14
N1	228	8	25	7	29	104	22	0	3	11	5	0	0
N2	10	10	134	40	238	62	19	1	24	1	27	1	0
N3	11	124	4	3	21	3	211	0	1	34	37	0	0
N4	0	1	2	0	4	5	8	63	1	4	0	5	0
N5	2	5	1	10	32	17	2	0	0	3	0	66	2
N6	3	2	91	661	4	2	0	0	0	4	3	2	3
N7	1	10	0	4	0	3	73	1	0	175	0	0	1
N8	18	1	619	171	8	42	6	0	0	7	1	4	1
N9	3	12	9	12	9	3	8	0	2	5	144	1	0

## Data Availability

The data that support the findings of this study are available from the corresponding author upon reasonable request.

## Supplementary Materials

The following supporting information can be downloaded at: https://www.mdpi.com/article/10.3390/ijms24033020/s1.
