# Supplementary material for: Evolutionary Dynamics of Avian Influenza Viruses Isolated from Wild Birds in Moscow
Source: Int J Mol Sci. 2023 Feb 3;24(3):3020. doi: 10.3390/ijms24033020 (PMC9917497; doi:10.3390/ijms24033020)
Supplement: Supplementary file 1 [file ijms-24-03020-s001.zip › Figure captions.pdf]

Figure S1. Evolutionary tree of the HA H4 gene of Eurasian viruses;
Figure S2. Evolutionary tree of the NA N6 gene of Eurasian viruses;
Figure S3. Evolutionary tree of the PB2 gene of Eurasian H4N6 viruses;
Figure S4. Evolutionary tree of the HA H4 gene of American viruses;
Figure S5. Evolutionary tree of the NA N6 gene of American viruses;
Figure S6. Evolutionary tree of the PB2 gene of American H4N6 viruses;
Figure S7. Evolutionary tree of the HA H3 gene of Eurasian viruses;
Figure S8. Evolutionary tree of the NA N8 gene of Eurasian viruses;
Figure S9. Evolutionary tree of the PB2 HA gene of Eurasian H3 viruses.
