# Supplementary material for: Evolutionary Dynamics of Avian Influenza Viruses Isolated from Wild Birds in Moscow
Source: Int J Mol Sci. 2023 Feb 3;24(3):3020. doi: 10.3390/ijms24033020 (PMC9917497; doi:10.3390/ijms24033020)
Supplement: Supplementary file 1 [file ijms-24-03020-s001.zip › Figure S5 Evolutionary tree of the NA N6 gene of American viruses.pdf]

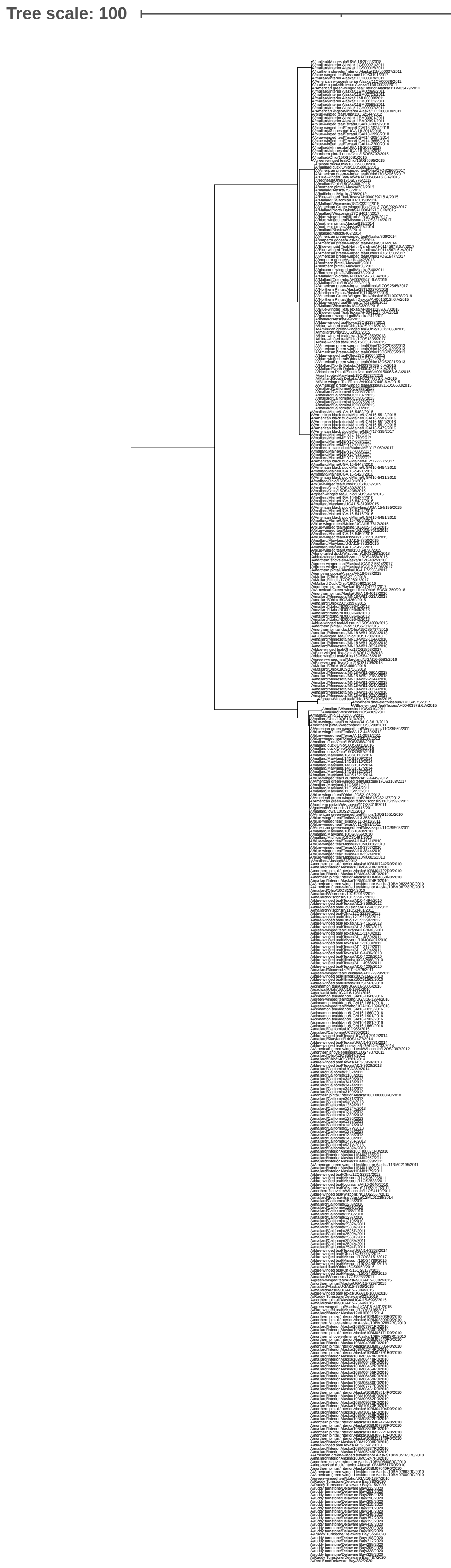
Tree scale: 100
