# Supplementary figures and images for: Evolutionary Dynamics of Avian Influenza Viruses Isolated from Wild Birds in Moscow
Source: Int J Mol Sci. 2023 Feb 3;24(3):3020. doi: 10.3390/ijms24033020 (PMC9917497; doi:10.3390/ijms24033020)

Tree scale: 0.1

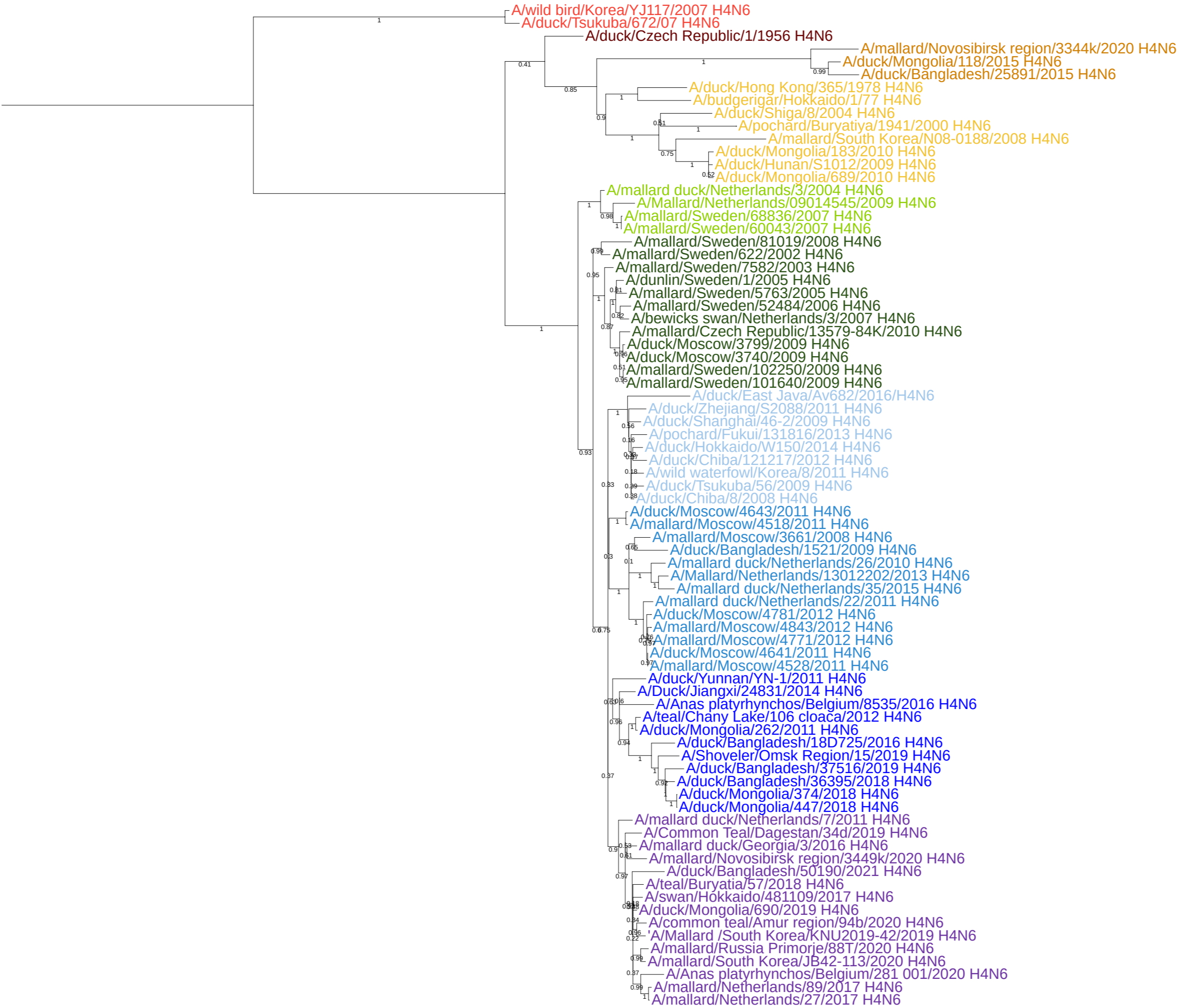

Supplement: Supplementary file 1 [file ijms-24-03020-s001.zip › Figure S1 Evolutionary tree of the HA H4 gene of Eurasian viruses.pdf]

Tree scale: 0.1

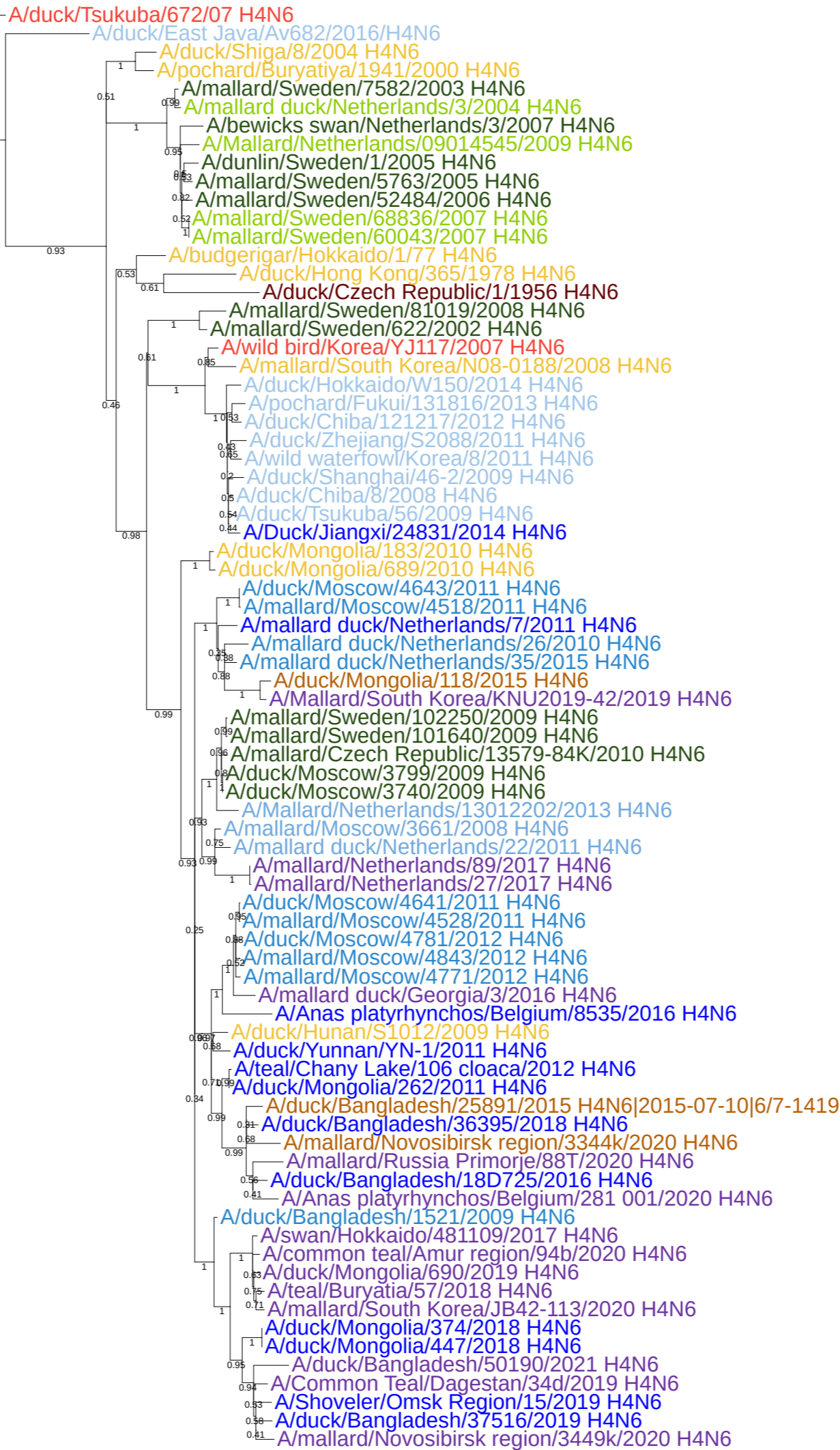

Supplement: Supplementary file 1 [file ijms-24-03020-s001.zip › Figure S2 Evolutionary tree of the NA N6 gene of Eurasian viruses.pdf]

Tree scale: 0.1

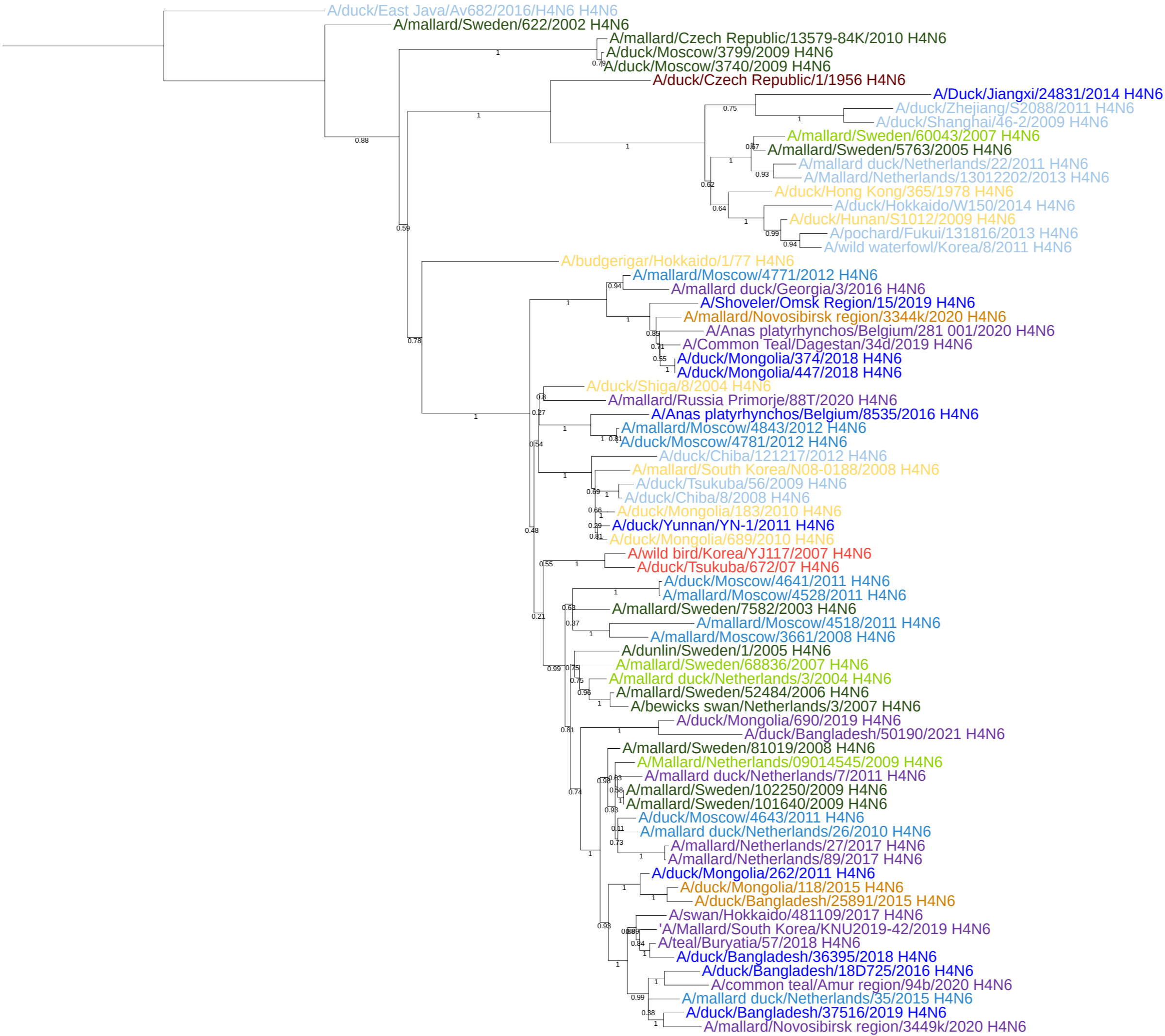

Supplement: Supplementary file 1 [file ijms-24-03020-s001.zip › Figure S3 Evolutionary tree of the PB2 gene of Eurasian H4N6 viruses.pdf]

Tree scale: 100

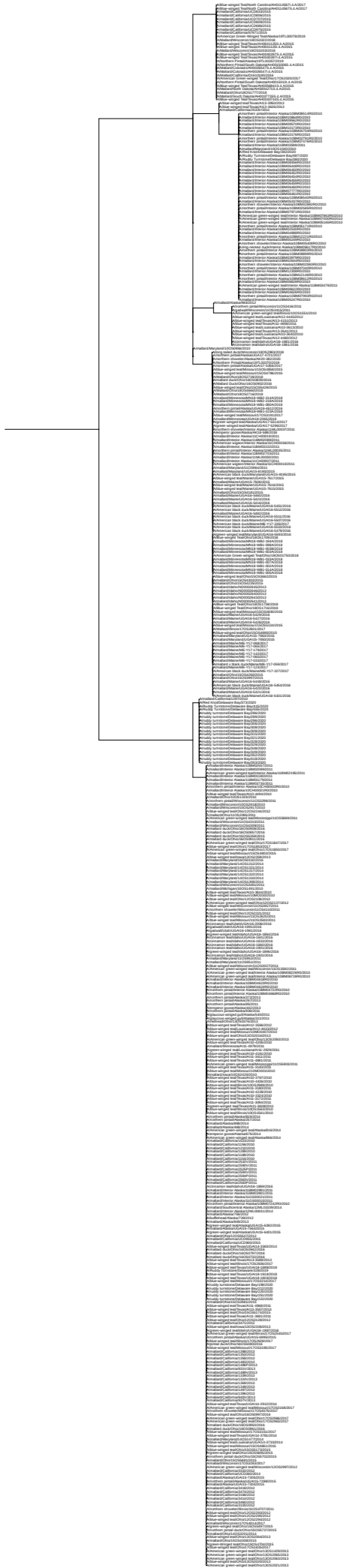

Supplement: Supplementary file 1 [file ijms-24-03020-s001.zip › Figure S4 Evolutionary tree of the HA H4 gene of American viruses.pdf]

Tree scale: 0.1

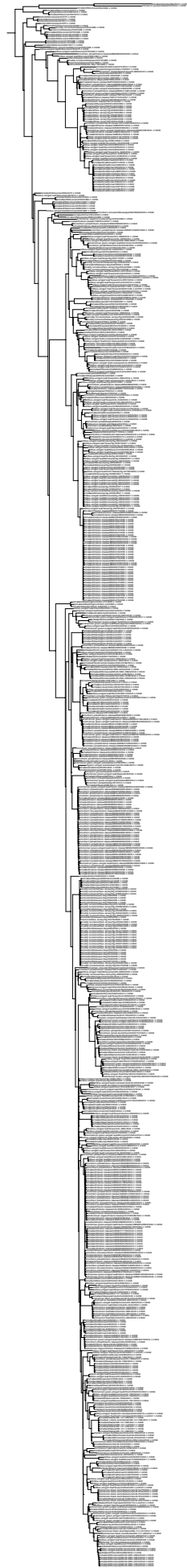

Supplement: Supplementary file 1 [file ijms-24-03020-s001.zip › Figure S6 Evolutionary tree of the PB2 gene of American H4N6 viruses.pdf]

Tree scale: 0.01

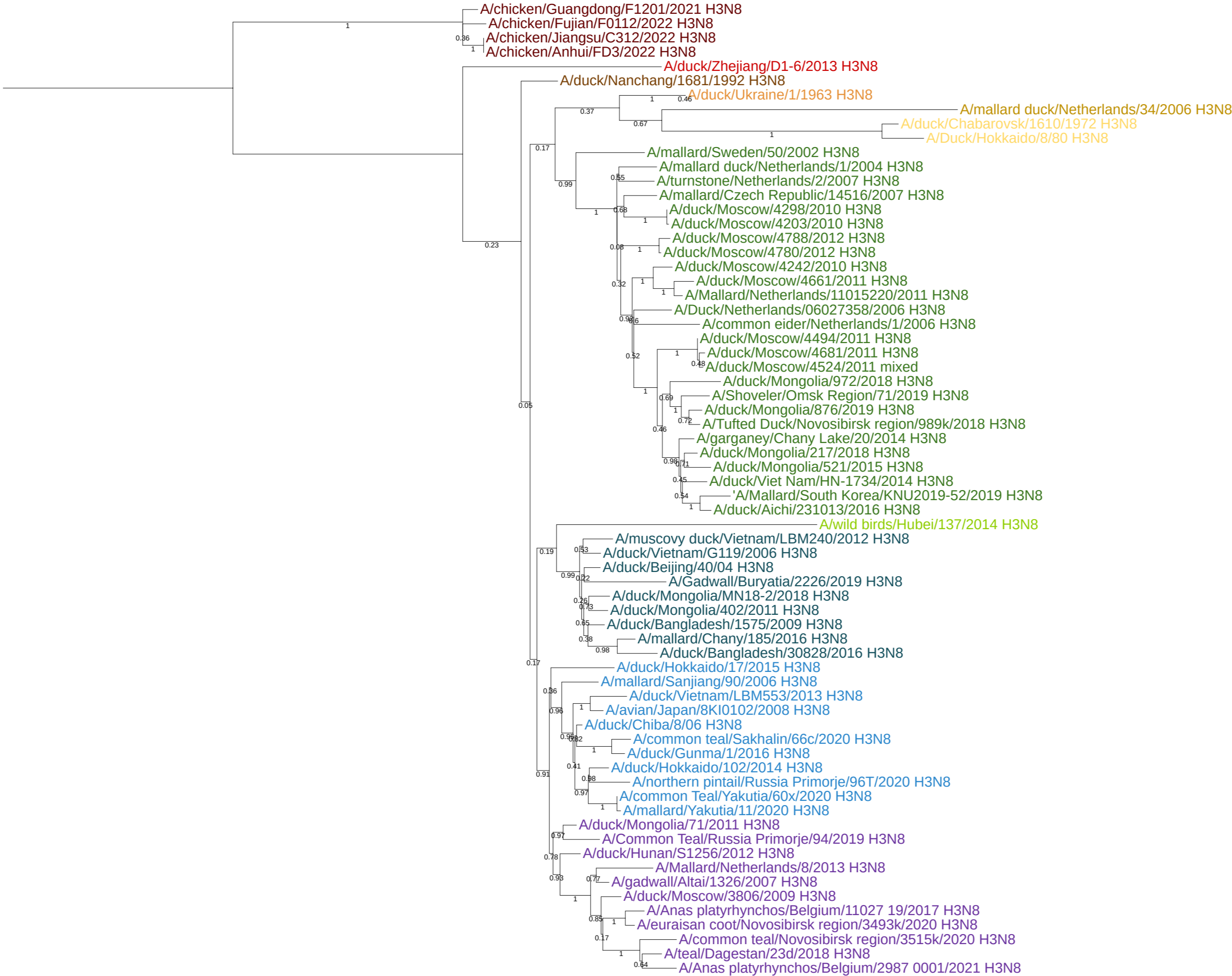

Supplement: Supplementary file 1 [file ijms-24-03020-s001.zip › Figure S7 Evolutionary tree of the HA H3 gene of Eurasian viruses.pdf]

Tree scale: 0.1

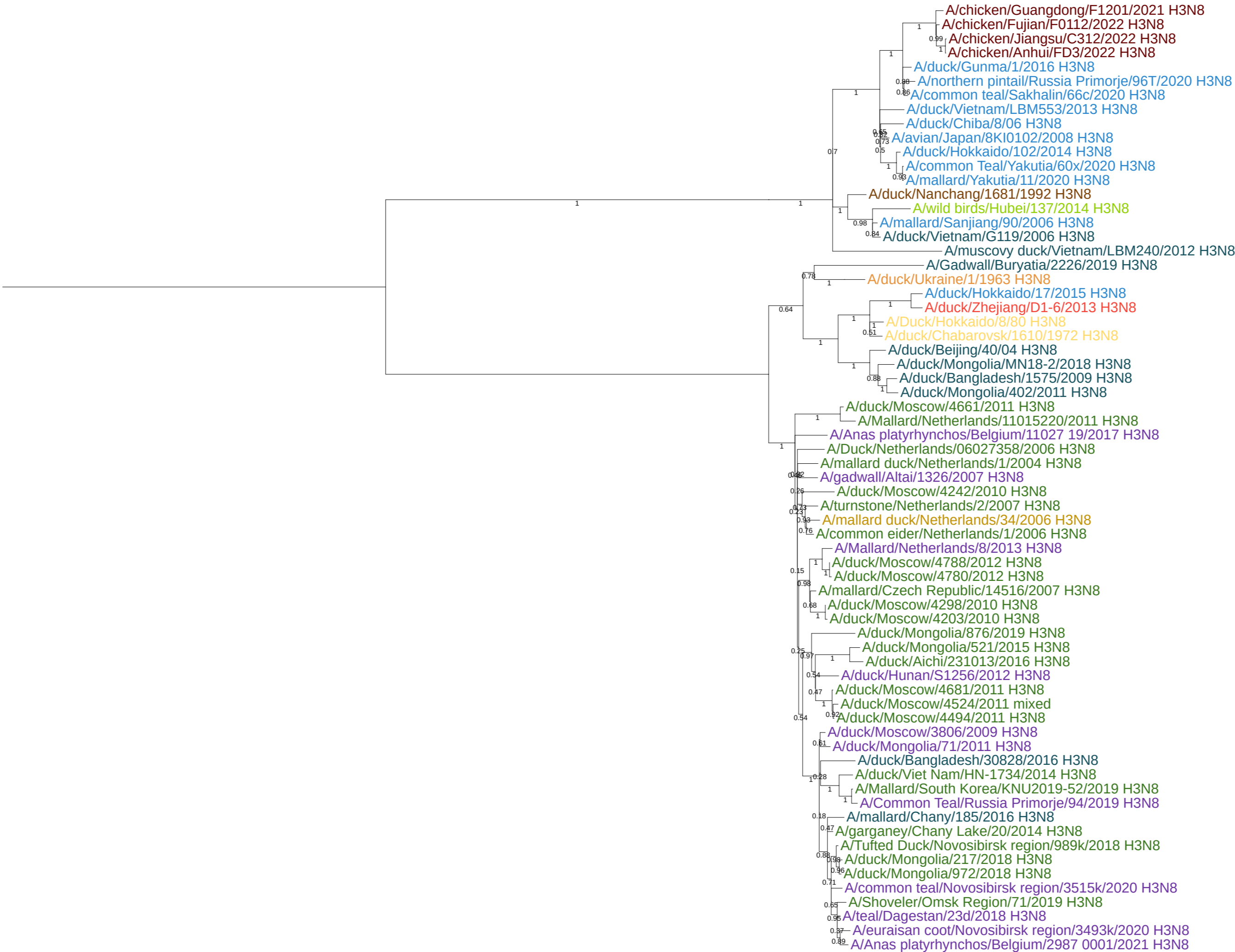

Supplement: Supplementary file 1 [file ijms-24-03020-s001.zip › Figure S8 Evolutionary tree of the NA N8 gene of Eurasian viruses.pdf]

Tree scale: 0.1

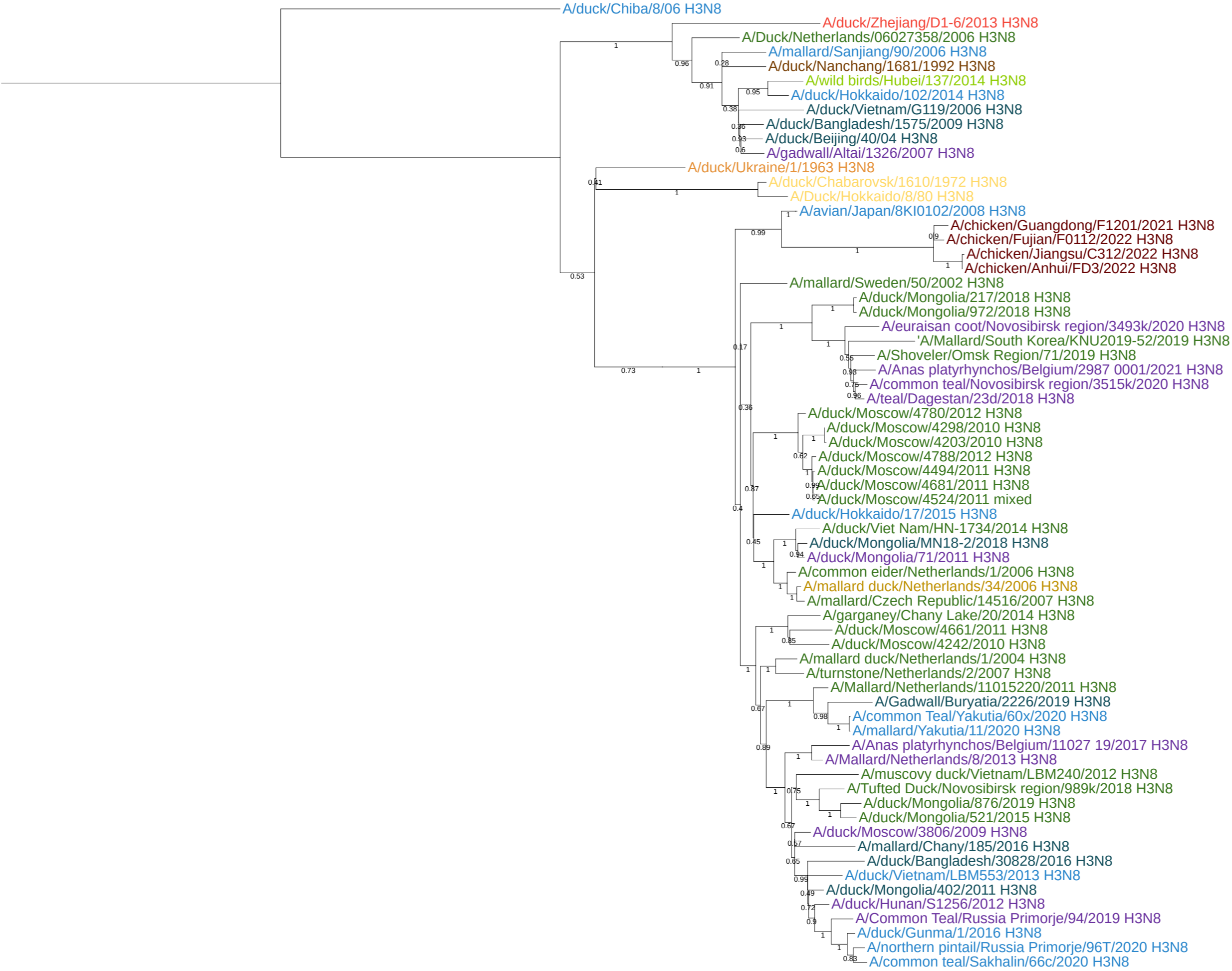

Supplement: Supplementary file 1 [file ijms-24-03020-s001.zip › Figure S9 Evolutionary tree of the PB2 HA gene of Eurasian H3 viruses.pdf]
